# Circulating genotypes of human papillomavirus in adult women of reproductive age from the Boeny region of Madagascar: a cross-sectional study to explore needs and opportunities for HPV vaccination in the country

**DOI:** 10.1186/s12879-026-12691-2

**Published:** 2026-01-28

**Authors:** Ekaterine Garsevanidze, Irina Kislaya, Jean-Marc Kutz, Tahinamandranto Rasamoelina, Sonya Ratefiarisoa, Ravo Razafindrakoto, Zaraniaina Tahiry Rasolojaona, Nantenaina Mathieu Razafindralava, Olivette Totofotsy, Alexina Olivasoa Zafinimampera, Tiana Randrianarisoa, Myriam Lassmann, Aaron Remkes, André Brito, Diavolana Koecher Andrianarimanana, Rivo Solotiana Rakotomalala, Zoly Rakotomalala, Pia Rausche, Jana Hey, Sandrine McKay-Chopin, Jürgen May, Monika Hampl, Valentina Marchese, Tarik Gheit, Rivo Andry Rakotoarivelo, Daniela Fusco

**Affiliations:** 1 Institute of International Health, Charité Center for Global Health (CCGH), Berlin, Germany; 2 RG Implementation Research, Bernhard Nocht Institute for Tropical Medicine, Bernhard- Nocht-Str. 74, D-20359 Hamburg, Germany; 3 Department of Infectious Diseases Epidemiology, Bernhard Nocht Institute for Tropical Medicine, Hamburg, Germany; 4 German Center for Infection Research (DZIF), Hamburg-Borstel-Lübeck- Riems, Germany; 5 Centre d’Infectiologie Charles Mérieux, Antananarivo, Madagascar; 6 University Fianarantsoa, Fianarantsoa, Madagascar; 7 Centre Hospitalier Universitaire Androva, Mahajanga, Madagascar; 8 International Agency for Research on Cancer (IARC), Lyon, France; 9 Tropical Medicine I, University Medical Center Hamburg-Eppendorf (UKE), Hamburg, Germany; 10 Köln-Hohenlind Hospital, Cologne, Germany

**Keywords:** HPV, Madagascar, Prevalence, Genotypes, Vaccination programs

## Abstract

**Background:**

HPV is the most common sexually transmitted infection worldwide, related to the vast majority of cervical cancers (CC). Prevention through vaccination against HPV is considered one of the most effective control measures for CC. Madagascar is one of the few countries where HPV vaccination has not yet been widely disstrivuted in the country as the national program just started in December 2025. This study aims to estimate the prevalence of HPV genotypes in rural Madagascar in order to support the planning of a national vaccine implementation strategy.

**Methods:**

A cross-sectional study was conducted between 2021 and 2022 in the Boeny region of Madagascar, involving women 18 to 49 years old. Cervico-vaginal lavages were collected and analysed for the presence of HPV DNA, covering 21 HPV genotypes. The prevalence of each HPV genotype was calculated as proportions with 95% confidence intervals (CI95%).

**Results:**

From a total of 927 women enrolled, 44.6% (*n* = 413, CI95%: 41.3, 47.8) tested positive for HPV. The most commonly detected high-risk genotypes were HPV52 8.3% (CI95%: 6.6, 10.3), HPV45 7.2% (CI95%: 5.6, 9.1), and HPV51 4.8% (CI95%: 3.5, 6.3). The prevalence of vaccine-target genotypes was 7.4% (CI95%: 5.8, 9.3), 10.8% (CI95%: 8.9, 13.0), and 29.3% (CI95%: 26.4, 32.4) for 2-valent, 4-valent, and 9-valent vaccines, respectively.

**Conclusions:**

Our results suggest that for a vaccination strategy aimed at preventing CC in Boeny, adoption of the 9-valent vaccine would increase the impact of the program.

**Clinical trial:**

Not applicable.

**Supplementary Information:**

The online version contains supplementary material available at 10.1186/s12879-026-12691-2.

## Background

Human Papillomavirus (HPV) is one of the most common viruses worldwide, with over 200 different types divided into five major genera: alpha, beta, gamma, mu, and nu [1]. It infects the basal keratinocytes of the mucosal and cutaneous epithelia, leading to common dermatologic diseases and various cancers [2–4]. Globally, it is the most prevalent sexually transmitted infection (STI), but can also be transmitted through skin contact [5, 6].

HPV types are commonly classified into high (HR- carcinogenic) or low-risk (LR- non-carcinogenic) [1]. Twelve alpha mucosal HPV types (HPV 16, 18, 31, 33, 35, 39, 45, 51, 52, 56, 58, and 59), referred to as high-risk HPV types (HR-HPV), were classified as carcinogenic to humans. Eight other alpha HPV types (HPV 26, 53, 66, 67, 68, 70, 73, and 82) were classified as probably or possibly carcinogenic (pHR-HPV) [7]. Cervical Cancer (CC) is the most frequent HPV-attributable cancer and is almost always associated with HR-HPV [2]. Mixed infections frequently occur, and it has been hypothesised that co-infection with LR-HPV is associated with a lower risk of future invasive disease and longer time to diagnosis than infection with HR-HPV alone [8].

In 2006, the first HPV vaccines became available, offering protection against the strains most likely to cause genital warts or CC [9]. There are currently six licensed HPV vaccines: three 2-valent (HPV16, 18), two 4-valent (HPV16, 18, 6, 11), and one 9-valent (HPV16, 18, 6, 11, 31, 33, 45, 52, 58); five are prequalified by the World Health Organisation (WHO) [9]. Real-world evidence suggests that vaccination could considerably reduce the incidence of HPV-related precancerous lesions and CC by 70–90% [10]. The 2-valent and 4-valent vaccines specifically target the two HR-HPV genotypes (16 and 18), which cause approximately 77% of CC worldwide, while the 9-valent vaccine targets seven HR genotypes, which account for approximately 94% of CC globally [3]. Additionally, the 4-valent and 9-valent vaccines provide protection against two LR-HPV genotypes (6 and 11), responsible for approximately 90% of genital warts [11]. The immunisation schedule is being debated globally: initially the WHO recommended the use of a three-dose regimen, followed by shift to two doses, but at the moment, many countries are shifting towards a single dose schedule due to the increasingly convincing data on immunogenicity and effectiveness together with ease of implementation and vaccine shortages [9, 12].

In November 2020, the WHO launched a global initiative to eliminate CC as a public health problem, aiming to vaccinate at least 90% of girls against HPV by the age of 15 years, to screen 70% of women using a high-performance test by the age of 35 years and again by the age of 45, and to treat at least 90% of identified precancerous lesions and invasive cancers [13]. However, the implementation of HPV vaccination and population-based screening programmes in low-resource settings, particularly in Sub-Saharan Africa (SSA), has been challenging due to financial, technological, logistical, and socio-cultural factors [14]. At present, 147 out of 194 WHO countries have included HPV vaccination programs within the national immunisation plans [15]. Madagascar is among the countries in which HPV vaccinations was not routinely implemented until December 2025, despite the fact that HPV prevalence is relatively high compared to other countries in SSA and, according to the few studies available, lies at over 36% [16]. As a result of high prevalence of HPV infection, CC is the most common cancer in Madagascar [4]. While worldwide there has been a trend towards the decrease of cases, in Madagascar the CC incidence remains high (41.2 per 100,000 women), 10 times higher than the WHO targets [13], mostly due to the scarcity or almost absence of any preventive measures. Madagascar is a country with a fragile health system in which both women’s health and cancer care suffer from implementation gaps, resulting in poor health outcomes and a poor prognosis for women diagnosed with CC [17]. The beginning of the immunization program represents a first step towards a concrete fight agains CC in the country. Though, Madagascar exhibits one of the lowest overall routine vaccination rates worldwide [18]. Complex political and policy developments, limited infrastructural capacity, as well as financial and operational challenges, are some of the main reasons for Madagascar’s low vaccination coverage [19, 20].

In view of the ongoing implementation of the HPV national vaccination program, a deep understanding of the HPV epidemiology in the country is critical to support the planning of the interventions. In this view, with the present study, we aimed at providing evidence for the conceptualisation of an HPV vaccination program in the country. The objective of the study was to measure the prevalence of the circulating types and describe the rate and type of co-infections so as to define the most effective vaccine to introduce on the basis of the circulating types among adult women of reproductive age.

## Methods

### Study design

This cross-sectional study is a secondary analysis of data collected between 2021 and 2022 at three Primary Health Care Centres (PHCCs) in the Boeny region of Madagascar [21]. The catchment area of these three PHCCs includes around 60,000 inhabitants. The PHCCs of Ankazomborona (16°06′50′′′S, 46°45′24′′E) and Antanambao Andranolava (15°58′00′′′S, 46°41′00′′′E) are considered rural areas, and Marovoay-Ville (16°06′40′′′S, 46°38′38′′E) as peri-urban.

### Eligibility criteria

Women aged between 18 and 49 years old who were resident in the region, fluent in Malagasy or French, and provided informed consent were eligible to participate. Pregnancy at the time of recruitment was the exclusion criterion. Women who did not provide a cervico-vaginal lavage (CVL) sample or had invalid HPV genotyping results (beta-globin = 0) were excluded from the analysis.

### Sampling

We used a non-probabilistic sampling strategy. Following outreach activities in the study catchment area, interested women who complied with the eligibility criteria were invited for a gynaecological examination at selected PHCCs. Participants were recruited on a first-come, first-served basis.

The sample size calculations were performed using the “*presize”* package of R software [22]. Given the low expected genotype-specific prevalence of HPV (below 10% [16]), we followed a precision requirement recommended in the literature, ensuring the width of the 95% confidence interval (CI95%) did not exceed the point estimate [23]. Assuming that the expected genotype-specific prevalence of HPV would range between 2% and 10%, a sample of at least 860 participants was required to estimate the exact CI95% with the desired precision.

### Data and sample collection

Participant recruitment took place from March 2021 to December 2022. The interviews, gynaecological examinations and CVL sample collection were conducted by six midwives, trained to ensure standardisation and accuracy of study procedures. Socio-demographic data were collected using a paper-based questionnaire previously described in Kutz et al. [21]. Each participant was assigned a unique identifier to ensure anonymisation. Data was entered into a REDCap-based database [24] using a double-entry procedure. Quality control of data processing and validation was performed regularly during and after data entry. To collect CVL, physiological fluid was applied to the vaginal wall and cervix and then collected back into a syringe while swabbing and brushing the cervix. A 10 ml sample from CVL was collected from all participants in liquid-based cytology medium containing tubes (ThinPrep, Hologic, Marlborough, Massachusetts, USA). Samples were stored at the central laboratory of Antananarivo (*Centre d’Infectiologie Charles Merieux*) before being shipped to the International Agency for Research on Cancer (IARC) in Lyon, France, where samples were analysed using a type-specific PCR bead-based multiplex genotyping assay (E7-MPG) that combines multiplex PCR and Luminex technology (Luminex Corp., Austin, TX, USA), as previously described by Schmitt et al. [25]. The assay detects twelve HR-HPV types (HPV 16, 18, 31, 33, 35, 39, 45, 51, 52, 56, 58, 59), seven pHR-HPV types (HPV 26, 53, 66, 68a and b, 70, 73, 82), and two low-risk types (HPV 6 and 11), with beta-globin amplification included as a DNA quality control. After multiplex PCR, products were denatured and hybridized to bead-coupled probes in 96-well plates, washed, and stained with a streptavidin-phycoerythrin conjugate. Beads were then analysed on a Luminex reader to identify HPV types and quantify fluorescence. Results were expressed as median fluorescence intensity (MFI) from at least 100 beads per type, with positivity defined using a cut-off based on background MFI values [25]. Women who were infected with more than one of the 21 genotypes were classified as having multiple infections.

### Statistical analysis

Data analyses were conducted using R (v.4.4.1). Participants’ characteristics were summarised using frequencies and percentages. The prevalence of genotype-specific HPV, single and multiple infections, as well as LR-HPV, pHR-HPV, and HR-HPV and vaccine-targeted genotypes, were presented as proportions. Corresponding CI95% were estimated using the Clopper-Pearson method. Prevalence estimates of any HPV infection, HR-HPV, LR-HPV, and multiple infections were stratified by age groups and urbanisation. The frequency of co-occurrence of multiple genotypes was described in the form of a heatmap.

To evaluate the potential impact of HPV vaccine introduction in the region, we analysed the distribution of infections caused by vaccine-target genotypes among HPV-positive women by age group and urbanisation. Cases with infection by HPV 6, 11, 16 or 18 without the presence of any other HPV genotype were considered potentially preventable by the 4-valent vaccine, while cases infected with HPV 31, 33, 45, 52, 58 either alone or in co-infections with HPV 6, 11, 16, 18, without the presence of any other HPV genotype were considered preventable by 9-valent vaccine. The Sison and Glaz method was used to estimate CI95% [26]. The chi-square test was used for the group comparisons. The statistical significance level of 5% was considered.

## Results

### Participants characteristics

Overall, 1035 women aged between 18 and 49 years were recruited for the study, 500 in 2021 and 535 in 2022; of those, 16 (1.5%) were excluded due to failure to collect a CVL sample and 92 (8.9%) were excluded after quality control (beta-globin = 0) (Figure S1). Characteristics of participants excluded from the analysis are reported in the supplementary material (Table S1).

Among 927 women included in the analysis, 36.1% were < = 25 years old, 61.5% were living in rural areas, and 12.2% had no formal education, while 43.3% reported secondary school or above (Table 1). None of the participants were vaccinated against HPV.

**Table 1:** Sociodemographic characteristics and clinical history of the study participants

Characteristic	*n* (%)
Urbanisation (*N* = 927)	
Rural	570 (61.5%)
Peri-urban	357 (38.5%)
**Age group **(*N* = 927)	
<=25y	335 (36.1%)
26-35y	318 (34.3%)
>=36y	274 (29.6%)
**Education level **(*N* = 927)	
No formal education	113 (12.2%)
Primary	413 (44.6%)
Secondary or higher	401 (43.3%)

### HPV prevalence by age group and urbanisation

**Fig. 1:**
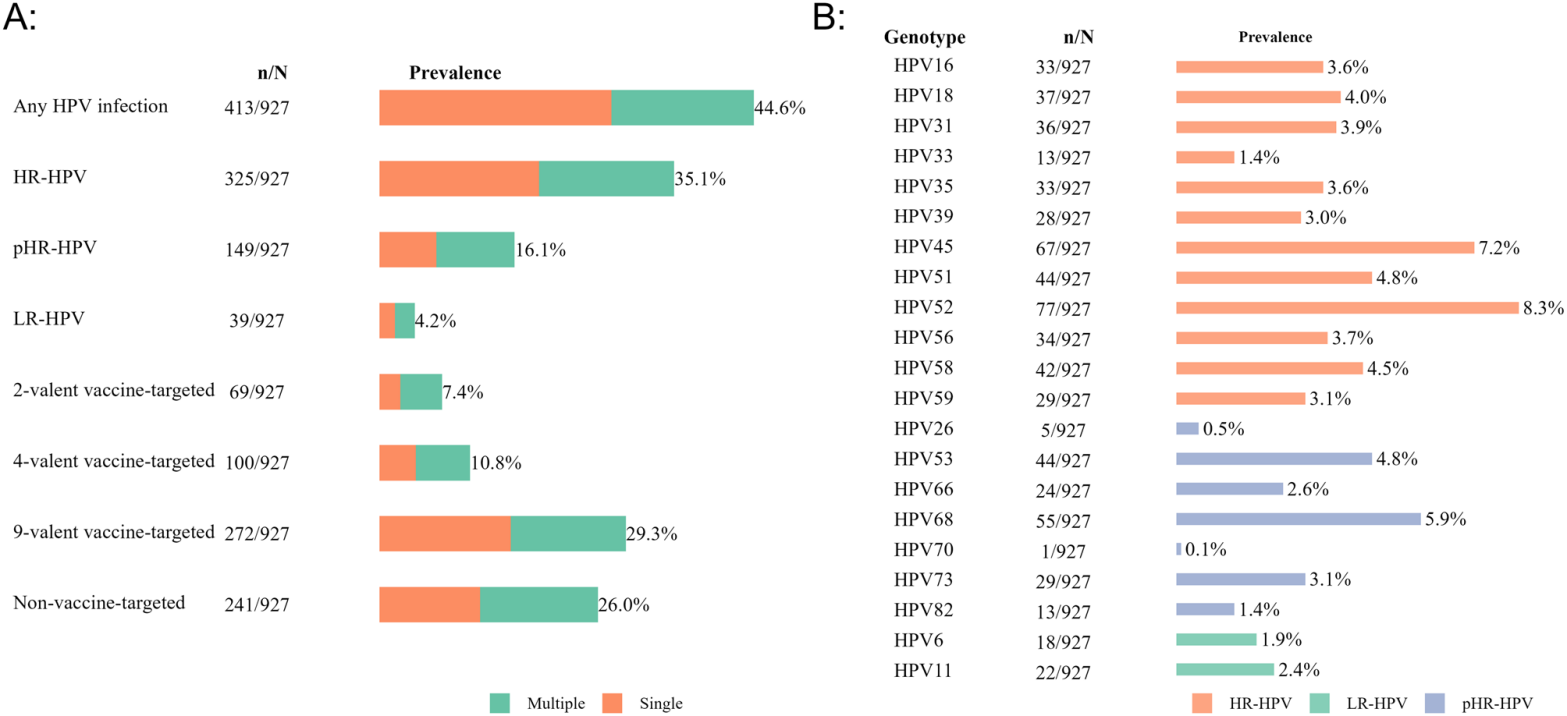
Prevalence (%) of HPV genotypes in Boeny, Madagascar, 2021–2022. Note: Panel **A**: Prevalence (%) of any HPV, HR-HPV, pHR-HPV, LR-HPV, vaccine-target and non-vaccine target HPV genotypes. Panel **B**: Genotype-specific HPV prevalence (%)

Of the 927 women included in the final analysis, 44.6% (*n* = 413, CI95%: 41.3, 47.8) tested positive for HPV. The prevalence of any HPV infection was 49.0% (*n* = 175, CI95%: 43.7, 54.3) in peri-urban areas and 41.8% (*n* = 238, CI95%: 37.7, 45.9) in rural areas, respectively (Fig. 1A, Table S2). Prevalence of HPV infection varied by age group, declining from 54.9% (*n* = 184, CI95%: 49.4, 60.3) among women < = 25 years old to 39.1% (*n* = 107, CI95%: 33.2, 45.1) among those aged > = 36 years old.

Infections with HR-HPV (35.1%, *n* = 325, CI95%: 32.0, 38.2) were more prevalent in the study population, compared to pHR-HPV, 16.1% (*n* = 149, CI95%: 13.8, 18.6) and LR-HPV genotypes, 4.2% (*n* = 39, CI95%: 3.0, 5.7), respectively. The HR-HPV prevalence declined from 44.2% to 27.7% with increasing age (Fig. 1A, Table S2), no differences in HR-HPV prevalence were observed between rural and peri-urban settings.

The prevalence of genotypes covered by the HPV vaccines was 7.4% (*n* = 69, CI95%: 5.8, 9.3), 10.8% (*n* = 100, CI95%: 8.9, 13.0), and 29.3% (*n* = 272, CI95%: 26.4, 32.4) for 2-valent, 4-valent, and 9-valent vaccines, respectively (Fig. 1A, Table S2).

### Genotype-specific HPV prevalence

The genotype-specific HPV prevalence varied from 0.1% (*n* = 1, CI95%: 0.0, 0.6) to 8.3% (*n* = 77, CI95%: 6.6, 10.3) (Fig. 1B, Table S3). The five most prevalent HR-HPV genotypes were HPV52 8.3% (*n* = 77, CI95%: 6.6, 10.3), HPV45 7.2% (*n* = 67, CI95%: 5.6, 9.1), HPV51 4.8% (*n* = 44, CI95%: 3.5, 6.3), HPV58 4.5% (*n* = 42, CI95%: 3.3, 6.1), and HPV18 4.0% (*n* = 37, CI95%: 2.8, 5.5). Among pHR-HPV genotypes, HPV68 had the highest prevalence 5.9% (*n* = 55, CI95%: 4.5, 7.7), while LR-HPV genotypes were detected with similar frequency (HPV11 2.4%, *n* = 22, CI95%: 1.5, 3.6; HPV6 1.9%, *n* = 18, CI95%: 1.2, 3.1) (Fig. 1B, Table S3).

### Single and multiple HPV infections

A single genotype was detected in 27.6% (*n* = 256, CI95%: 24.8, 30.6) of participants, while 16.9% (*n* = 157, CI95%: 14.6, 19.5) were infected with multiple genotypes. The maximum number of genotypes detected per HPV-positive sample was 8.

**Fig. 2:**
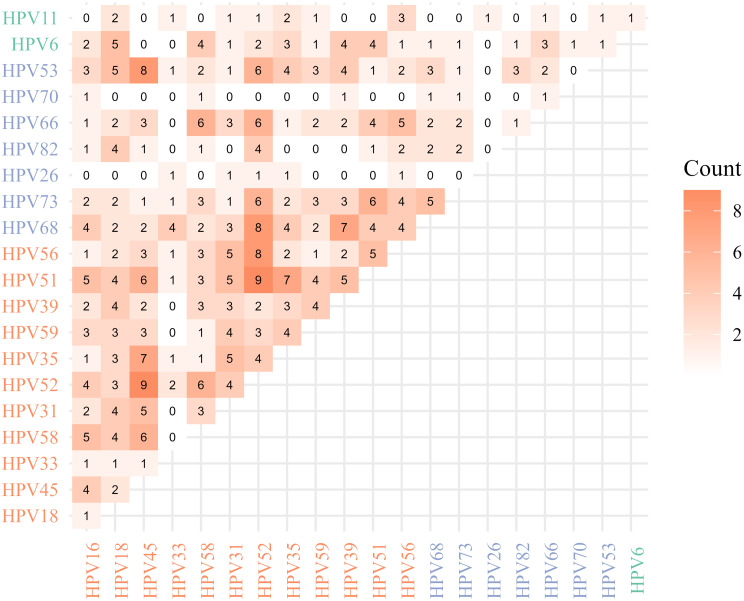
Frequency of co-occurrence of HPV genotype-specific infections in Boeny, Madagascar, 2021–2022. Note: HR-HPV in orange, pHR-HPV in blue and LR-HPV in green

Of the 157 women with multiple genotype infections, 59.9% (*n* = 94) had dual infections, while 20.4% (*n* = 32) had triple genotype infections (Table S4). The most common co-infection patterns observed in our study included a combination of HR-HPV with pHR-HPV genotypes (45.2%, *n* = 71), followed by co-infection with different HR-HPV genotypes (36.3%, *n* = 57), and a combination of HR-HPV with LR-HPV genotypes (8.9%, *n* = 14). HPV52 (*n* = 42, 26.8%), HPV45 (*n* = 33, 21.0%) and HPV51 (*n* = 30, 19.1%) were specific genotypes most frequently involved in multiple infections (Table S3). Figure 2 illustrates the co-occurrence of genotype-specific infections. The most common combinations were HPV52 with HPV45 and HPV52 with HPV51, which occurred 9 times, followed by the combinations of HPV52 with HPV56, HPV52 with HPV68, and HPV45 with HPV53, which occurred 8 times.

### HPV infections potentially preventable with vaccination

**Fig. 3:**
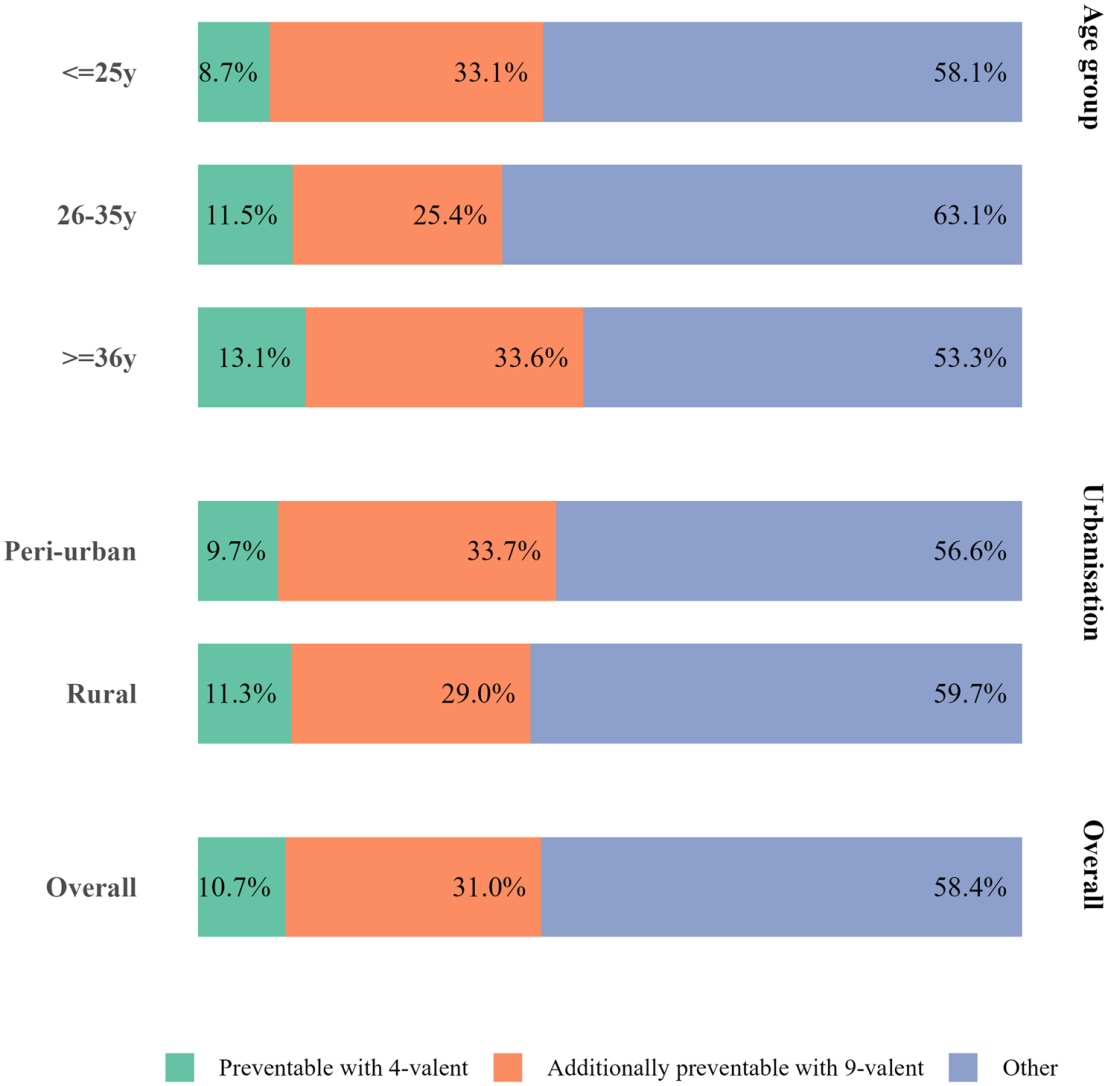
Distribution of HPV infections associated with vaccine target genotypes by age group and urbanisation

Among 413 women who tested positive for HPV, 41.6% (*n* = 172, CI95%: 36.8, 46.6) were infected exclusively by vaccine-targeted genotypes, indicating potential for a considerable reduction of HPV infections with vaccination. Namely, 10.7% (*n* = 44, CI95%: 5.8, 15.6) of infections could be prevented with the 4-valent vaccine, while the 9-valent vaccine had an incremental preventive benefit of 31.0% (*n* = 128, CI95%: 26.2, 35.9) (Fig. 3). We observed no statistically significant differences in the distribution of potentially preventable infections by age group (p-value = 0.402) or level of urbanisation (p-value = 0.565) (Table S5).

## Discussion

This study shows that in the Boeny region of Madagascar, there is a high prevalence of HR-HPV (35.1%) genotypes among adult women of reproductive age. Additionally, it shows that 41.6% of the infections could be preventable by vaccination, which has been just implemented in the country. Finally, more than one-third of co-infections are between HR and HR strains (36.3%), suggesting a high potential to progress towards more severe forms of CC.

Our data show that HPV prevalence in Boeny, Madagascar is high (44.6%) when compared to other studies on the general female population in SSA countries, with a conserved trend in the proportion of HR infection [27].

The most common genotypes identified in our study are 52 (8.3%), 45 (7.2%), 51 (4.8%), 58 (4.5%) and 18 (4.0%), showing a very unique typing profile of the region if compared to the global frequencies or previous studies in Madagascar [16]. In fact, the prevalence of HPV16, the most common genotype worldwide [28], which solely accounts for 63.7% of squamous cell carcinomas and 46.4% of adenocarcinomas [3], was relatively low in the population under study (3.6%), compared to other HR-HPV genotypes covered by the 9-valent vaccine. Interestingly, the third most common genotype identified in Madagascar is HPV51, which is not covered by any available vaccine. HPV51 has been classified as HR carcinogenic since 2005, but is considered to have lower carcinogenic potential than HPV 16 and HPV18, with around 0.5% of CC globally attributable to HPV51 [3]. However, in Africa, HPV51 has frequently been described in co-infections in CC cases [29]. High prevalence of this genotype has been previously reported in unvaccinated women in Wales [30], Brazil [31], China [32], and, following the implementation of vaccination programs, in Germany [33] and the USA [34]. These findings are particularly important and show the relevance for monitoring and comparison of circulating types before and after vaccinations, as it is unknown whether genotype replacement will occur in the long-term following vaccination, with mathematical modelling suggestive of this hypothesis [35]. Although evidence shows that in the majority of countries, including the SSA region, in which vaccines were introduced at least eight years ago, a decrease in HPV prevalence has been observed [36, 37] not enough data are available so far regarding the circulating genotypes before and after vaccination, especially in SSA [38]. In December 2025 Madagascar officially introduced HPV vaccination into the national immunization schedule [39]. This study will provide a unique opportunity to monitor changes in the prevalence of the virus and different genotypes over time after the implementation of vaccination.

Our data show age-related patterns in the prevalence of HPV infection, including vaccine-target genotypes, and some disparities between rural and peri-urban communities. The HPV prevalence was the highest in the < = 25 years old group and subsequently declined with age. This trend is in line with what has been described globally, that HPV prevalence peaks among young adults following sexual debut and declines with age, reaching a plateau around 40–50 years [28]. The real-world data and modelling studies suggest that “catch-up” and multi-cohort vaccination could considerably increase the impact of the immunisation program [40]. In a context of relatively late age of sexual debut (median = 17.1 years) [41], adopting multi-age cohort vaccination strategies could accelerate the reduction of HPV infections and HPV-related disease in young vulnerable populations.

In contrast with previous studies [16], our data show higher HPV prevalence in peri-urban settings (49.0 vs.41.8%). This result could be attributed to differences in social norms and greater women’s autonomy regarding sexual behaviours in peri-urban settings [41]. The differences in HPV prevalence between rural and peri-urban communities need to be monitored following vaccine rollout. Lower awareness of HPV in rural settings in Madagascar and more barriers to healthcare use could endanger equitable access to vaccines as well as uptake, which is essential for a successful vaccination campaign [20]. This study provides, for the first time, robust estimates of HPV prevalence and an assessment of the HPV genotypes in Boeny, Madagascar, based on samples and data collected via highly standardised procedures and through analysis performed at the IARC, which is the worldwide reference institution in the field of HPV. This data could support public health stakeholders in designing a HPV immunisation program in Madagascar. Despite these strengths, our study does not come without limitations. First, we used a convenience sampling strategy and recruited participants in PHCCs. Although this approach helped to reduce costs and provided all necessary conditions for study procedures, it could have led to some selection bias. Participants with higher literacy, who were more health-conscious, and who had better ability to access PHCCs due to logistical and cost factors might be overrepresented in our sample. Second, the exclusion of initially recruited participants due to failure to obtain a CVL sample (1.5%) or to quality control issues (8.9%) may also introduce selection bias. However, the absence of statistically meaningful differences between included and excluded women on key demographic variables suggests a low risk of bias from this exclusion. Third, our study had limited statistical power for a reliable comparison of genotype-specific HPV prevalence across population subgroups and analysis of co-infection patterns and specific genotypes involved in triple and multiple infections. Estimates based on ≤ 5 positive cases should be interpreted with caution. Furthermore, the assay used in our study targeted 21 HPV genotypes; this could limit the comparability of our findings to other studies based on different assays. Additionally, our analysis was restricted to circulating genotypes and did not relate genotype distribution to cytopathological findings, such as cervical lesions, cervical intraepithelial neoplasia, or invasive cancer. Further studies that focus on HPV genotypes in women with cervical pathology are necessary in Madagascar. Lastly, for the calculation of potentially preventable infections, we assumed vaccine effectiveness of 100% and no cross-protection.

## Conclusions

In conclusion, our study reports a high prevalence of HR-HPV genotypes targeted by the 9-valent vaccine in Madagascar’s Boeny region, supporting national public health authorities in the choice of vaccine products and planning of an HPV immunisation campaign. We believe that with the implementation of tailored HPV vaccination strategies, Madagascar could reduce HPV circulation and CC incidence in the next years. At the same time, a clear strategy for CC prevention overall is poorly implemented in the country and its adoption might be critical in case of type replacement and increase of genotypes not currently covered by vaccines. In fact, following the recent HPV vaccination startegy roll-out in Madagascar, this study further gives opportunity of monitoring the circulating HPV genotypes before and after vaccination in order to mitigate the consequences of type-replacement in case it would arise. We advocate for an increase in local laboratory capacities for HPV testing for sustainable surveillance of circulating genotypes, which is necessary for vaccine program evaluation.

## Supplementary Information

Below is the link to the electronic supplementary material.

Supplementary Material 1

## Data Availability

The datasets analysed during the current study so as the HPV typing data are available from the corresponding author on reasonable request, and will be freely available to researchers who wish to use them for non-commercial purposes, without breaching the confidentiality of participants.

## Abbreviations

CC: Cervical cancer
CI95%: Confidence interval 95%
CVL: Cervico-vaginal lavage
DNA: Deoxyribonucleic acid
GAVI: Global Alliance for Vaccines and Immunisation
HPV: Human Papillomavirus
HR: High-risk
IARC: International Agency for Research on Cancer
LR: Low-risk
PCR: Polymerase Chain Reaction
pHR: Probable high-risk
PHCC: Primary Health Care Centre
REDCap: Research Electronic Data Capture
SSA: Sub-Saharan Africa
WHO: World Health Organisation
