# Supplementary material for: Circulating genotypes of human papillomavirus in adult women of reproductive age from the Boeny region of Madagascar: a cross-sectional study to explore needs and opportunities for HPV vaccination in the country
Source: BMC Infect Dis. 2026 Jan 28;26:425. doi: 10.1186/s12879-026-12691-2 (PMC12924319; doi:10.1186/s12879-026-12691-2)

**Additional file 1**

Circulating genotypes of human papillomavirus in adult women of reproductive age from the Boeny region of Madagascar: a cross-sectional study to explore needs and opportunities for HPV vaccination in the country

**Table S1. Distribution of included and excluded individuals by key demographic variables**

| **Characteristic** | Included  n (%) | Excluded  n(%) | p-value^1^ |
| --- | --- | --- | --- |
| **Urbanisation** |  |  | 0.939 |
| Rural | 570 (61.5%) | 66 (61.1%) |  |
| Peri-urban | 357 (38.5%) | 42 (38.9%) |  |
| **Age group** |  |  | 0.051 |
| <=25y | 335 (36.1%) | 52 (48.2%) |  |
| 26-35y | 318 (34.3%) | 30 (27.8%) |  |
| >=36y | 274 (29.6%) | 26 (24.0%) |  |
| **Education level** |  |  | 0.363 |
| No formal education | 113 (12.2%) | 15 (13.9%) |  |
| Primary | 413 (44.6%) | 54 (50.0%) |  |
| Secondary or higher | 401 (43.3%) | 39 (36.1%) |  |

^1^The distribution of key characteristics of included participants and those excluded due to failure to collect a CVL sample or after quality control (beta-globin=0) was compared using chi-square tests.

**Table S2.** Prevalence of HPV infection among 18-49 years old women resident in the Boeny region, Madagascar stratified by age group and level of urbanisation

|  | N | n | Prevalence [CI95%] | p-value |
| --- | --- | --- | --- | --- |
| **Any HPV** | 927 | 413 | 44.6[41.3, 47.8] |  |
| Urbanisation |  |  |  | 0.030 |
| Peri-urban | 175 | 357 | 49.0[43.7, 54.3] |  |
| Rural | 238 | 570 | 41.8[37.7, 45.9] |  |
| Age group |  |  |  | <0.001 |
| <=25y | 335 | 184 | 54.9[49.4, 60.3] |  |
| 26-35y | 318 | 122 | 38.4[33.0, 44.0] |  |
| >=36y | 274 | 107 | 39.1[33.2, 45.1] |  |
| **HR-HPV** | 927 | 325 | 35.1[32.0, 38.2] |  |
| Urbanisation |  |  |  | 0.094 |
| Peri-urban | 357 | 137 | 38.4[33.3, 43.6] |  |
| Rural | 570 | 188 | 33.0[29.1, 37.0] |  |
| Age group |  |  |  | <0.001 |
| <=25y | 335 | 148 | 44.2[38.8, 49.7] |  |
| 26-35y | 318 | 101 | 25.2[20.5, 30.3] |  |
| >=36y | 274 | 76 | 17.9[13.5, 22.9] |  |
| **Probable HR-HPV** | 927 | 149 | 16.1[13.8, 18.6] |  |
| Urbanisation |  |  |  | 0.113 |
| Peri-urban | 357 | 66 | 18.5[14.6, 22.9] |  |
| Rural | 570 | 83 | 14.6[11.8, 17.7] |  |
| Age group |  |  |  | 0.116 |
| <=25y | 335 | 65 | 19.4[15.3, 24.1] |  |
| 26-35y | 318 | 45 | 14.2[10.5, 18.5] |  |
| >=36y | 274 | 39 | 14.2[10.3, 18.9] |  |
| **LR-HPV** | 927 | 39 | 4.2[3.0, 5.7] |  |
| Urbanisation |  |  |  | 0.742 |
| Peri-urban | 357 | 16 | 4.5[2.6, 7.2] |  |
| Rural | 570 | 23 | 4.0[2.6, 6.0] |  |
| Age group |  |  |  | 0.050 |
| <=25y | 335 | 21 | 6.3[3.9, 9.4] |  |
| 26-35y | 318 | 8 | 2.5[1.1, 4.9] |  |
| >=36y | 274 | 10 | 3.7[1.8, 6.6] |  |
| **Multiple HPV infections** | 927 | 157 | 16.9[14.6, 19.5] |  |
| Urbanisation |  |  |  | 0.414 |
| Peri-urban | 357 | 65 | 18.2[14.3, 22.6] |  |
| Rural | 570 | 92 | 16.1[13.2, 19.4] |  |
| Age group |  |  |  | 0.004 |
| <=25y | 335 | 72 | 21.5[17.2, 26.3] |  |
| 26-35y | 318 | 54 | 17.0[13.0, 21.6] |  |
| >=36y | 274 | 31 | 11.3[7.8, 15.7] |  |
| **2-valent (16, 18)** | 927 | 69 | 7.4 [5.8, 9.3] |  |
| Urbanisation |  |  |  | 0.255 |
| Peri-urban | 357 | 31 | 8.7[6.0, 12.1] |  |
| Rural | 570 | 38 | 6.7[4.8, 9.0] |  |
| Age group |  |  |  | 0.125 |
| <=25y | 335 | 28 | 8.4[5.6, 11.9] |  |
| 26-35y | 318 | 28 | 8.8[5.9, 12.5] |  |
| >=36y | 274 | 13 | 4.7[2.6, 8.0] |  |
| **4-valent (16, 18, 6, 11)** | 927 | 100 | 10.8[8.9, 13.0] |  |
| Urbanisation |  |  |  | 0.232 |
| Peri-urban | 357 | 44 | 12.3[9.1, 16.2] |  |
| Rural | 570 | 56 | 9.8[7.5, 12.6] |  |
| Age group |  |  |  | 0.120 |
| <=25y | 335 | 45 | 13.4[10.0, 17.6] |  |
| 26-35y | 318 | 32 | 10.1[7.0, 13.9] |  |
| >=36y | 274 | 23 | 8.4[5.4, 12.3] |  |
| **9-valent (16, 18, 6, 11, 31, 33, 45, 52, 58)** | 927 | 272 | 29.3[26.4, 32.4] |  |
| Urbanisation |  |  |  | 0.016 |
| Peri-urban | 357 | 121 | 33.9[29.0, 39.1] |  |
| Rural | 570 | 151 | 26.5[22.9, 30.3] |  |
| Age group |  |  |  | 0.027 |
| <=25y | 335 | 116 | 34.6[29.5, 40.0] |  |
| 26-35y | 318 | 86 | 27.0[22.2, 32.3] |  |
| >=36y | 274 | 70 | 25.6[20.5, 31.1] |  |
| **Non-vaccine target** | 927 | 241 | 26.0[23.2, 28.9] |  |
| Urbanisation |  |  |  | 0.341 |
| Peri-urban | 357 | 99 | 27.7[23.2, 23.7] |  |
| Rural | 570 | 142 | 24.9[21.4, 28.7] |  |
| Age group |  |  |  | 0.005 |
| <=25y | 335 | 107 | 31.9[27.0, 37.2] |  |
| 26-35y | 318 | 77 | 24.2[19.6, 29.3] |  |
| >=36y | 274 | 57 | 20.8[16.2, 26.1] |  |

p-value: p-value of the Chi-square test

**Table S3** Type-specific prevalence of HPV infection among 18-49 years old women resident in the Boeny region, Madagascar

| Type | n | % | CI95% | Single  n (%)^1^ | Multiple  n (%)^2^ |
| --- | --- | --- | --- | --- | --- |
| **HR-HPV** |  |  |  |  |  |
| HPV16 | 33 | 3.6 | [2.5, 5.0] | 12 (4.7%) | 21(13.4%) |
| HPV18 | 37 | 4.0 | [2.8, 5.5] | 11 (4.3%) | 26 (16.6%) |
| HPV31 | 36 | 3.9 | [2.7, 5.3] | 15 (5.9%) | 21 (13.4%) |
| HPV33 | 13 | 1.4 | [0.7, 2.4] | 4 (1.6%) | 9 (5.7%) |
| HPV35 | 33 | 3.6 | [2.5, 5.0] | 6 (2.3%) | 27 (17.2%) |
| HPV39 | 28 | 3.0 | [2.0, 4.3] | 6 (2.3%) | 22 (14.0%) |
| HPV45 | 67 | 7.2 | [5.6, 9.1] | 34 (13.3%) | 33 (21.0%) |
| HPV51 | 44 | 4.8 | [3.5, 6.3] | 14 (5.5%) | 30 (19.1%) |
| HPV52 | 77 | 8.3 | [6.6, 10.3] | 35 (13.7%) | 42(26.8%) |
| HPV56 | 34 | 3.7 | [2.6, 5.1] | 9 (3.5%) | 25 (15.9%) |
| HPV58 | 42 | 4.5 | [3.3, 6.1] | 17 (6.6%) | 25 (15.9%) |
| HPV59 | 29 | 3.1 | [2.1, 4.5] | 13 (5.1%) | 16 (10.2%) |
| **Probable** **HR-HPV** |  |  |  |  |  |
| HPV26* | 5 | 0.5 | [0.2, 1.3] | 2(0.8%) | 3(1.9%) |
| HPV53 | 44 | 4.8 | [3.5, 6.3] | 18(7%) | 26(16.6%) |
| HPV66 | 24 | 2.6 | [1.7, 3.8] | 7(2.7%) | 17(10.8%) |
| HPV68 | 55 | 5.9 | [4.5, 7.7] | 25(9.8%) | 30(19.1%) |
| HPV70* | 1 | 0.1 | [0.0, 0.6] | 0(0%) | 1(0.6%) |
| HPV73 | 29 | 3.1 | [2.1, 4.5] | 9(3.5%) | 20(12.7%) |
| HPV82 | 13 | 1.4 | [0.7, 2.4] | 2(0.8%) | 11(7%) |
| **LR-HPV** |  |  |  |  |  |
| HPV6 | 18 | 1.9 | [1.2, 3.1] | 3(1.2%) | 15(9.6%) |
| HPV11 | 22 | 2.4 | [1.5, 3.6] | 14(5.5%) | 8(5.1%) |

*≤5 positive cases used to estimate the prevalence

^1^ Percentages are calculated using number of women with single HPV infection (n=256) as the denominator

^2^ Percentages are calculated using the number of women with multiple HPV infections (n=157) as the denominator

**Table S4. Characterisation of HPV infection with multiple genotypes among HPV positive**

|  | N | n | %[CI95%] | | |
| --- | --- | --- | --- | --- | --- |
| HR+pHR | 157 | 71 | 45.2[37.3, 53.4] | | |
| HR+HR | 157 | 57 | 36.3[28.8, 44.4] | | |
| HR+LR | 157 | 14 | 8.9[5.0, 14.5] | | |
| pHR+pHR | 157 | 7 | 4.5[1.8, 9.0] | | |
| pHR+LR | 157 | 7 | 4.5[1.8, 9.0] | | |
| LR+pHR* | 157 | 1 | 0.6[0.0, 3.5] | | |
| Number of infections |  |  |  |  |  |
| 2 | 157 | 94 | 59.9[51.8, 67.6] | | |
| 3 | 157 | 32 | 20.4[14.4, 27.5] | | |
| 4 | 157 | 19 | 12.1[7.5,18.3] | | |
| 5 | 157 | 7 | 4.5[1.8, 9.0] | | |
| 6+* | 157 | 5 | 3.2[1.0, 7.3] | | |

*≤5 positive cases used to estimate the prevalence

**Table S5** Distribution of vaccine-target HPV infections by age group and urbanisation among HPV-positive women

|  |  | Preventable  with 4-valent | Additionally preventable  with 9-valent | Non-vaccine target | p-value |
| --- | --- | --- | --- | --- | --- |
| **Urbanisation** |  |  |  |  | 0.565 |
| Peri-urban | n | 17 | 59 | 99 |  |
|  | %[CI 95%] | 9.7[2.3, 17.3] | 33.7[26.3, 41.3] | 56.6[49.1, 64.1] |  |
| Rural | n | 27 | 69 | 142 |  |
|  | %[CI 95%] | 11.3[5, 17.7] | 29[22.7, 35.3] | 59.7[53.4, 66] |  |
|  |  |  |  |  |  |
| **Age group** |  |  |  |  | 0.402 |
| <=25y | n | 16 | 61 | 107 |  |
|  | %[CI 95%] | 8.7[1.6, 16.1] | 33.2[26.1, 40.6] | 58.2[51.1, 65.6] |  |
| 26-35y | n | 14 | 31 | 77 |  |
|  | %[CI 95%] | 11.5[3.3, 20.3] | 25.4[17.2, 34.2] | 63.1[54.9, 71.9] |  |
| >=36y | n | 14 | 36 | 57 |  |
|  | %[CI 95%] | 13.1[3.7, 23.1] | 33.6[24.3, 43.6] | 53.3[43.9, 63.2] |  |
| Overall | n | 241 | 128 | 44 |  |
|  | %[CI 95%] | 58.4[53.5, 63.3] | 31[26.2, 35.9] | 10.7[5.8, 15.6] |  |

Figure S1: Participants' inclusion flow chart


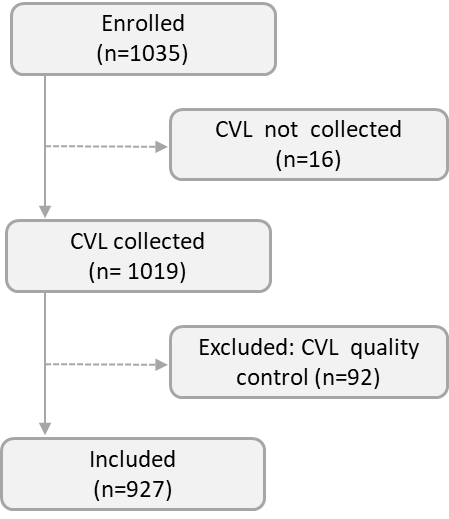

Supplement: Supplementary file 1 — Supplementary Material 1 [file 12879_2026_12691_MOESM1_ESM.docx]
